# AQP5 trafficking is regulated by its C-terminal tail and interaction with prolactin-inducible protein

**DOI:** 10.1186/s13062-025-00647-6

**Published:** 2025-04-16

**Authors:** Claudia D’Agostino, Egor Zindy, Louise Conrard, Amel Takkal, Françoise Gregoire, Nargis Bolaky, Susanna Törnroth-Horsefield, Jason Perret, Christine Delporte

**Affiliations:** 1 Laboratory of Pathophysiological and Nutritional Biochemistry, Université Libre de Bruxelles, Brussels, Belgium; 2 Center for Microscopy and Molecular Imaging (CMMI), Université Libre de Bruxelles, Gosselies, Belgium; 3 Division of Biochemistry and Structural Biology, Department of Chemistry, Lund University, Lund, Sweden

## Abstract

**Background:**

Aquaporin-5 (AQP5) is a crucial membrane protein involved in water transport across cellular membranes, particularly within exocrine glands such as salivary glands. Dysregulation of AQP5, including its mislocalization, has been associated with various diseases, emphasizing the need to understand the molecular mechanisms governing its trafficking. This study investigates the multifaceted regulatory mechanisms of AQP5 trafficking, with specific emphasis on the role of the carboxyl-terminal (C-terminal) tail and the functional involvement of prolactin-inducible protein (PIP) as an interacting protein partner.

**Methods:**

An innovative 2D-custom model employing SNAP-tag human AQP5 constructs together with a novel automated algorithm-based methodology was used following immunofluorescence and confocal microscopy to assess hAQP5 localization to the plasma membrane of stably transfected normal salivary gland-SV40 transformed-acinar cells (NS-SV-AC). The expression of the constructs was verified by Western blot analysis.

**Results:**

The expression of SNAP-hAQP5 constructs expressed in stably transfected NS-SV-AC cells allowed to explore the involvement of hAQP5 C-terminal tail and the hAQP5-hPIP interaction in hAQP5 trafficking upon stimulation. The use of C-terminal truncation constructs revealed distinct responses to intracellular 3’,5’-cyclic adenosine monophosphate (cAMP) and calcium increase, shedding light on the importance of specific regions within the highly flexible distal part of the C-terminal tail for AQP5 trafficking. Furthermore, our investigation of the interplay between hAQP5 and hPIP revealed that PIP promotes AQP5 translocation to the plasma membrane, blunting the effects of calcium- and cAMP-dependent pathways on AQP5 sub-cellular localization.

**Conclusion:**

In summary, this study advances our understanding of AQP5 trafficking dynamics and provides critical insights into the regulatory roles of the C-terminal tail and its interaction with PIP. The innovative methodology to assess AQP5 translocation to the plasma membrane sets the stage for future investigations to identify the role of individual amino acids and phosphorylation sites within the distal AQP5 C-terminus in the trafficking mechanism and protein-protein interaction, and to explore the dynamic of the process by high resolution live cell imaging. Further research in this area is warranted to uncover critical insights into the regulation of AQP5, offering opportunities for the development of innovative therapeutic strategies.

**Supplementary Information:**

The online version contains supplementary material available at 10.1186/s13062-025-00647-6.

## Introduction

Aquaporins (AQPs) are integral membrane proteins that play a pivotal role in the selective and rapid transport of water across cellular membranes [1]. In humans, thirteen isoforms of AQPs (AQP0-AQP12) have been characterized. AQP5 is detected in various tissues including cornea [2], lung, lacrimal glands and salivary glands [3] with a predominant localization on the apical membrane of acinar cells in exocrine glands following stimuli-induced trafficking to the plasma membrane [4]. The role of AQP5 in salivary glands has been revealed using *Aqp5*-knockout mice. In contrast to their wild-type counterparts, *Aqp5*-deficient mice exhibited a notable 60% reduction in saliva secretion upon pilocarpine stimulation, accompanied by increased saliva viscosity and hypertonicity [5]. These data highlighted the central role of AQP5 in preserving water balance and homeostasis. Dysregulation of AQP5 has been associated with various diseases, including Sjögren’s syndrome (SS) [6], bronchitis [7] and cystic fibrosis [8], stressing the clinical significance for understanding the molecular mechanisms governing AQP5 trafficking and function. The alteration of AQP5 function is particularly relevant in the context of xerostomia, characterized by oral dryness and often results from salivary gland hypofunction [9]. Xerostomia can be attributed to various causes including SS, genetic anomalies, endocrine or neurological disorders, and head and neck cancer radiotherapy [10].

A fundamental property of most human AQPs is their ability to be regulated by trafficking, which allows membrane water permeability to be controlled in response to cellular or environmental signals. This phenomenon has been best described for aquaporin 2 (AQP2) in the kidney collecting duct principal cells where AQP2 trafficking to the apical membrane in response to the pituitary hormone vasopressin underlies urine volume regulation. The AQP2 C-terminus plays a major role in AQP2 sub-cellular localization by harboring multiple post-translational modifications sites that act as sorting signals in the trafficking mechanism. Of these, Serine 256 (Ser256) is of particular importance, as phosphorylation of this site by protein kinase A (PKA) is necessary and sufficient for AQP2 to be targeted to the apical membrane [11]. The high sequence and structural homology between AQP2 and AQP5 C-terminal tails has raised the hypothesis that the AQP5 C-terminus also plays a crucial role in its regulation via similar mechanisms as those controlling trafficking of AQP2. Indeed, the C-terminal tail of AQP5 has been confirmed as a key element for governing its trafficking to the plasma membrane [12] using AQP5 chimeras [13] and constructs lacking the C-terminal domain [12]. AQP5 trafficking is governed by a complex interplay of regulatory pathways, with notable involvement of cAMP and calcium signaling pathways [14]. An increase in intracellular cAMP levels leads to both distinct short-term and long-term effects on AQP5 vesicular trafficking. Short-term exposure to cAMP triggers AQP5 internalization and lysosomal degradation through a mechanism involving PKA, whereas long-term exposure induces AQP5 translocation to the plasma membrane, accompanied by AQP5 phosphorylation [15]. Two potential PKA phosphorylation sites, Serine 156 (Ser156), located within loop D, and Threonin259 (Thr259), located at the C-terminus, play different roles in AQP5 trafficking [16]. Phosphorylation of Ser156, PKA and extracellular hypotonicity all independently increased AQP5 plasma membrane abundance levels, suggesting a dynamic interplay between multiple pathways [17]. For Thr259, which corresponds to Ser256 in AQP2, phosphorylation by the cAMP-PKA signaling pathway may play a role in channel gating [18] and is responsible for increased lateral diffusion of AQP5, i.e. localization within the plasma membrane [19]. However, the role of these phosphorylated amino acids on AQP5 trafficking remains to be fully determined [17, 18]. Calcium signaling is another crucial pathway involved in the regulation of AQP5 [20]. The activation of muscarinic M3 and α1 adrenergic receptors, leading to intracellular calcium increase, has been associated with AQP5 shuttling to the plasma membrane [21]. In addition, nitric oxide/cGMP signaling pathways have also been implicated in acetylcholine induced-AQP5 trafficking [20]. Despite the presence of several phosphorylation sites for protein kinase G (PKG) within AQP5, the extent of PKG involvement and the specific sites of phosphorylation await further elucidation. Moreover, the interactions between AQP5 and various protein partners, several of which interacting with the AQP5 C-terminal tail, may also play a crucial role in coordinating AQP5 trafficking and function in secretory cells [22]. AQP5 interacts with a variety of proteins such as Na-K-Cl cotransporter 1 (NKCC1), anion exchanger 2 (AE2) [23], Transient Receptor Potential Cation Channel Subfamily V Member 4 (TRPV4) [24], junctional proteins [25], Mucin 5AC (MUC5AC) [26], prolactin-inducible protein (PIP) [27], and ezrin [28]. The interplay between AQP5 and these protein partners plays a pivotal role in regulating its localization and function, effectively contributing to the preservation of distinctive physiological processes. Additionally, these interactions act as connectors between AQP5 and the actin cytoskeleton [29]. While the interaction between AQP5 C-terminus and PIP has already been documented by proximity ligation assay in transfected cells and in human minor salivary glands, as well as by microscale thermophoresis using recombinant proteins, the role of PIP on AQP5 translocation remains unknown [27].

The key function of AQP5 within exocrine glands and its implication in various diseases underscore the need to uncover the underlying mechanisms involved in its trafficking. The involvement of intracellular cAMP and calcium signaling pathways and the cytoskeleton, alongside interactions with protein partners, indicate the complex interplay that governs the role of AQP5 in health and disease. Further research is warranted to uncover critical insights into the regulation of AQP5, which may potentially lead to the development of innovative therapeutic strategies for related conditions. This study investigates the multifaceted regulatory mechanisms governing AQP5 trafficking, with a particular emphasis on the role of the C-terminal tail and the functional involvement of PIP as an interacting partner. To this end, we have generated constructs of full-length and C-terminally truncated human AQP5 with a self-labelling fluorescent tag (SNAP) fused to their N-terminus and developed a novel automated algorithm-based methodology for assessing their localization to the plasma membrane.

## Materials and methods

Cell culture.

Normal salivary gland-SV40 transformed-acinar cell line (NS-SV-AC, kindly donated by Professor Azuma, Second Department of Oral and Maxillofacial Surgery, Tokushima University School of Dentistry) [30] were grown, tested free of mycoplasma and authenticated as previously described [27].

### Design of plasmid vectors and stable transfection

Four plasmids were engineered to express a small protein tag derived from mammalian O6-alkylguanine-DNA-alkyltransferase (SNAP)-tagged added to the N-terminus of either the full-length wildtype human AQP5 (hAQP5) made of 265 amino acids (SNAP-hAQP5 (1-265)) or truncated C-terminal constructs: SNAP-hAQP5 (1-245), SNAP-hAQP5 (1-241) and SNAP-hAQP5 (1-227) (VectorBuilder, CA, USA) (Fig. S1A). The SNAP-tag was separated from the AQP5 coding sequences by a peptide spacer sequence comprising 15 amino acids equivalent to 3x repeats of the core linker sequence GGGGS (sourced from VectorBuilder, Chicago, IL, USA). The SNAP-tag is a compact protein with an approximate molecular weight of 19 kDa [31]. The plasmids use the strong EF1A promoter to drive the transcription of the recombinant SNAP-hAQP5 constructs. Five micrograms of plasmid were transfected by electroporation (270 V, 700 µF) into NS-SV-AC cells resuspended in Ingenio solution (Madison, WI, USA), using a Gene Pulser II System (Bio-Rad, Hercules, CA, USA). Twenty-four hours post transfection, stably transfected cells were selected for 7 days in complete medium supplemented with 5 µg/ml of puromycin (InvivoGen, San Diego, CA, USA), prior to performing limiting dilution cloning. A selected SNAP-hAQP5 (1-265) clone was additionally transfected with 5 µg of human PIP (hPIP) plasmid (VectorBuilder, Chicago, IL, USA) (Fig. S1B). Twenty-four hours post transfection, stable SNAP-hAQP5 (1-265)-PIP transfected cells were selected for 10 days in complete medium supplemented with 10 µg/ml of blasticidin (InvivoGen, San Diego, CA, USA).

### Western blot analysis

The expression of SNAP-hAQP5 constructs was assessed by Western blot (WB) analysis. Forty µg of protein underwent electrophoresis in either a Tris-Glycine 12% or 4-12% polyacrylamide gel before being transferred to a polyvinylidene fluoride (PVDF) membrane. Subsequently, the PVDF membranes were blocked for 1 h at room temperature (RT), using 10mM phosphate buffer saline (PBS) containing 0.1% Tween 20 (PBS-T) and 10% skimmed-milk. Following the blocking, the membranes were incubated overnight at 4 °C with PBS-T 5% skimmed-milk containing rabbit anti‐AQP5 (1:1000, AB15858, Sigma‐Aldrich, St Louis, MI, USA), SNAP-Tag Polyclonal Antibody (1 µg/ml, CAB4255, Invitrogen, Waltham, MA, USA), or mouse anti‐β‐actin clone C4 (MAB1501, Millipore, Temecula, CA, USA). After three washes with PBS-T, the membranes were incubated for 1h at RT with anti-rabbit horseradish peroxidase (HRP)-conjugated antibody diluted in PBS-T containing 5% skimmed milk. Next, the membranes were exposed to enhanced chemiluminescent (ECL) detection substrate, and immunoreactive proteins were visualized using the Amersham Imager 600 (GE Healthcare Life Sciences, Piscataway, NJ, USA).

### Immunofluorescence

Stable cell lines expressing SNAP-hAQP5 constructs or clone 3 NS-SV-AC cells expressing SNAP-hAQP5 (1-265) transfected without or with hPIP were seeded in 8-wells Millicell^®^ EZ slides (PEZGS0816; Merck Millipore, Burlington, MA, USA). The cells expressing SNAP-hAQP5 constructs were preincubated for 24 h with complete medium containing either no or 50 µM indomethacin (INDO; #I7378, Sigma-Aldrich, St Louis, MI, USA). Subsequently, cells were incubated for 8 h in the absence or presence of either 10µM forskolin (#1099, Tocris, Bristol, UK) [32, 33], 0.1µM thapsigargin (#1138, Tocris, Bristol, UK) [34], or a combination of both. To study hAQP5 or hPIP expression, all cells were fixed with 0.8% paraformaldehyde (PFA) for 20 min at RT, permeabilized with 0.1% Triton X-100 for 1 min and blocked with 10% normal donkey serum (AB 2337258, Jackson ImmunoResearch, West Grove, PA, USA) for 1 h at RT. Subsequently, the cells were incubated overnight at 4 °C with primary antibodies, either goat anti-AQP5 polyclonal antibodies (1:100, raised against the C-terminal 23 amino acids of hAQP5 and affinity-purified; Eurogentec, Seraing, Belgium), rabbit anti-SNAP-Tag polyclonal antibodies (1:1000; #CAB4255, Invitrogen, Waltham, MA, USA) or mouse anti-PIP (Abcam, Cambridge, UK). Then next day, the cells were incubated, for 1 h incubation at RT with secondary donkey AF594-conjugated anti-goat IgG (1:500; #A32758, Invitrogen, Waltham, MA, USA), goat AF594-conjugated anti-rabbit IgG (H + L), F(ab’)2 Fragment (1:500; #8889, Cell Signaling, Danvers, MA, USA) or goat FITC-conjugated anti-mouse IgG (H + L) highly cross-adsorbed secondary antibody (1:500; #A16079, Invitrogen, Waltham, MA, USA). Nuclei were stained using 1 µg/ml 4′,6-diamidino-2-phenylindole, dihydrochloride (DAPI). The slides were mounted using ProLong™ Gold Antifade Mountant (P36930, Thermo Fisher Scientific, Waltham, MA, USA) and #1.5 thickness coverslips. Fluorescence images of AQP5 labeling were acquired using an Axio Observer LSM710 equipped with lasers (a 405 nm diode for DAPI and a HeNe 594 nm laser for and AF594), a 40x/1.2 water immersion C-Apochromat objective, prism-based spectral separation and photomultiplier tubes detectors (adjusted for detection between 410 and 490 nm for DAPI and between 599 and 700 nm for AF594). A 1 AU pinhole aperture set on the 594 nm channel led to the acquisition of 1.1 μm section images with stack spacing of 0.54 μm, resulting in images with the following xyz scaling: 0.082*0.082*0.545 μm. Images were acquired using a pixel dwell of 0.50µsec, a line mean averaging of 2 and a bit depth of 16. Fluorescent images for hPIP labeling were acquired using a Leica DM microscope and a 20x objective.

### Image processing

Quantification of the SNAP-hAQP5 constructs labelling in the cell perimembrane area was performed on original images using CellProfiler software (Broad Institute, Cambridge, MA, USA), applying consistent parameters across all conditions. Our analysis is based on a partial segmentation of the cell membrane, focusing on fluorescent regions, to measure the intensity of the fluorescence emitted in the perimembrane area (Fig. S2). This measurement is performed on the acquisition channel corresponding to the detection of the SNAP-hAQP5 protein and corresponds to the upper quartile intensity, defined as the pixel intensity value below which 75% of the pixels in the object have lower values. This method was chosen over measuring the average intensity due to its reduced susceptibility to the influence of background pixels, outliers, and variations in intensity that can compromise the accuracy of measurements. To mitigate non-specific signals and eliminate perinuclear fluorescence, the fluorescence of the SNAP-hAQP5 protein localized at the cell nuclei was masked by using a segmentation of the nuclei on a separate DAPI channel and expanding their surface area. The expansion diameter was estimated based on multiple images to mask perinuclear fluorescence without affecting membrane signal detection. Object selection was based on measures of ‘area’ and ‘circularity’ (only long and thin objects representing membrane fragments were retained, excluding non-specific objects). The intensity of each perimembrane fragment was recorded in a CellProfiler database and processed using a custom Python script (available upon request; utilizing Pandas for data handling and Seaborn for visualization) which grouped the perimembrane fragments measurements from multiple images and across each condition, followed by exportation into an Excel file for further analysis.

### Statistical analysis

The data were analyzed using GraphPad Prism software version 10.4.1. (San Diego, CA, USA) using the non-parametric Kruskal-Wallis test with post-hoc Dunnet tests. Results were represented as Violin plots with median and interquartile range (IQR 25-75%) and considered statistically significant at *p* < 0.05.

## Results

### Expression of SNAP-tagged hAQP5 in NS-SV-AC cells

**Fig. 1:**
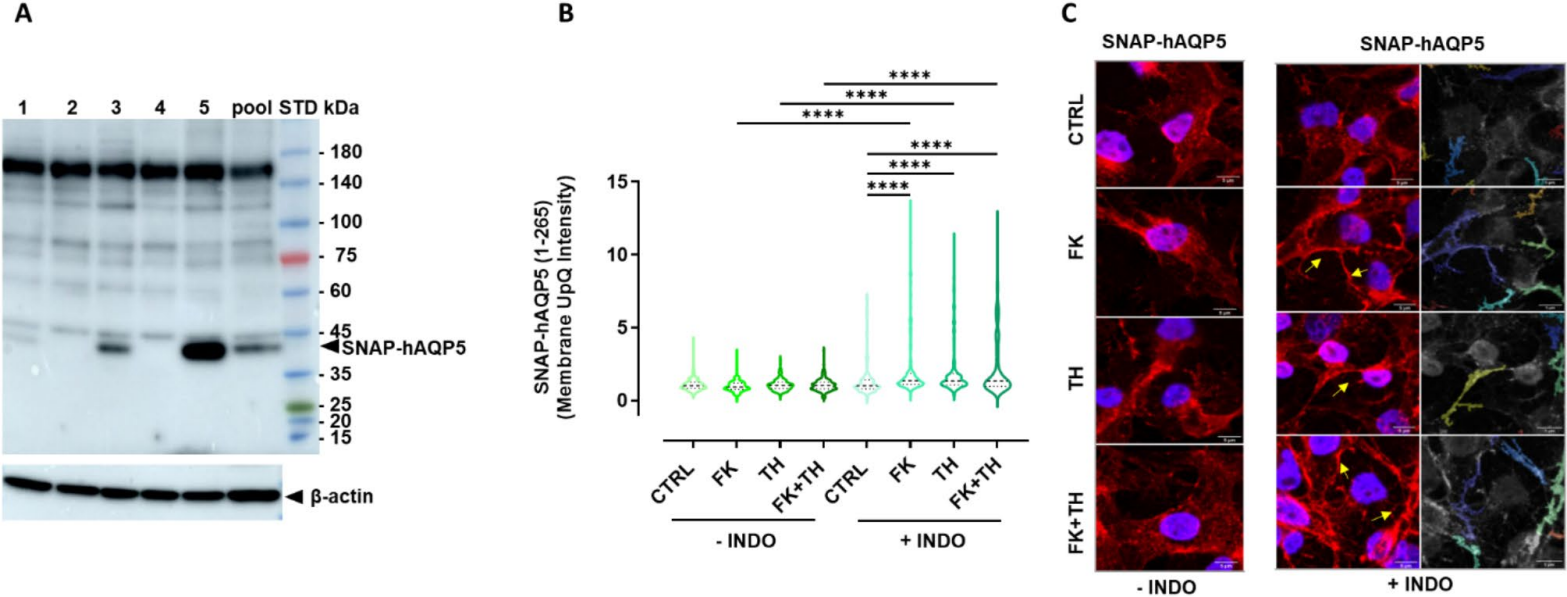
Expression and trafficking of SNAP-hAQP5 construct in NS-SV-AC cells. **(A)** WB analysis for AQP5 (using anti-AQP5 antibodies) and β-actin in five clones (1–5) or pool (used as positive control prior to limiting dilution cloning) of NS-SV-AC cells stably transfected with SNAP-hAQP5. STD: molecular weight standards. **(B-C)** Cells (clone 3 of NS-SV-AC SNAP-hAQP5) were pretreated for 24 h without or with 50µM indomethacin (-INDO: +INDO) prior to treatment for 8 h without (CTRL) or with 10 µM FK, 0.1 µM TH, or both (FK + TH), in 3 independent experiments. **(B)** Violin plots (with median and interquartile range of 25–75% percentile) of the membrane upper quartile (UpQ) intensity. Statistical significance evaluated using Kruskal-Wallis test with post-hoc Dunn’s tests is indicated as ****: *p* < 0.0001. In the absence of INDO pretreatment, the medians with IQR are: 1 with IQR 0.821–1.240, *n* = 203 cells for CTRL; 0.913 with IQR 0.755–1.180, *n* = 191 cells for FK; 1.027 with IQR 0.796–1.266, *n* = 229 cells for TH; 1.018 with IQR 0.780–1.265, *n* = 209 cells for FK + TH. In the presence of INDO pretreatment, the medians with IQR are: 1 with 0.789–1.414, *n* = 304 cells for CTRL; 1.347 with IQR 1.108–1.849, *n* = 414 for FK; 1.331 with IQR 1.066–1.816, *n* = 414 for TH; 1.331 with IQR 0.963–2.709, *n* = 410 for FK + TH. **(C)** Representative confocal images of immunofluorescent staining with anti-AQP5 antibodies (red) and DAPI (blue); yellow arrows indicate AQP5 localization close to the plasma membrane. Membrane segmentation images are also provided for cells pretreated with INDO. The specificity of the anti-AQP5 antibodies and the negative control performed in the absence of primary antibody are shown in Fig. S6

Non-transfected NS-SV-AC cells were used as negative controls (CTRL) due to their lack of detectable endogenous hAQP5 expression (no detectable AQP5-immunoreactive band at 23 kDa). NS-SV-AC cells transfected with the full length untagged hAQP5 (1-265; used as a positive control) exhibited an AQP5-immonoreactive band at approximately 23 kDa. NS-SV-AC stably transfected with SNAP-tagged full-length hAQP5 revealed an AQP5-immunoreactive band of approximately 42 kDa, displaying the anticipated molecular weight for the tagged protein (19 kDa SNAP-tag + 23 kDa AQP5) (Fig. S3). Following limiting dilution cloning, five clones were tested for the expression of SNAP-hAQP5: only clones 1, 3 and 5 showed AQP5-immunoreactive bands at approximately 42 kDa with variable intensities (with the lowest expression in clone 1 and the highest expression in clone 5) (Fig. 1A). The positive control, the SNAP-tagged hAQP5-pool (selected transfected cells before cloning), showed an AQP5-immunoreactive band of expected size (42 kDa). Similar results were obtained when Western blot was performed using anti-SNAP antibody (Fig. S4). Based on the moderate expression of SNAP-hAQP5 in clone 3, this clone was selected for all further experiments.

### Trafficking of SNAP-tagged hAQP5 in NS-SV-AC cells

The trafficking dynamics of the SNAP-hAQP5, expressed in the stably transfected NS-SV-AC clone 3 cells, was assessed. The cells were subjected to 24 h preincubation with or without 50µM indomethacin (INDO; an inhibitor of prostaglandin synthesis and prostaglandins-induced cAMP and calcium increase) to set all the cells in the culture to a similar basal intracellular level of cAMP and calcium. Then, the cells were incubated for 1 h and 8 h without (CTRL) or with 10µM forskolin (FK; that increases the second messenger cAMP), or 0.1µM thapsigargin (TH; antagonizing sarcoplasmic/endoplasmic reticulum Ca2+-ATPase (SERCA) pump that induces calcium release from the endoplasmic reticulum), or with a combination of both (FK + TH). After 8 h, but not after 1 h (Fig. S5), the preincubation with INDO significantly enhanced the subsequent response to stimuli inducing hAQP5 trafficking (Fig. 1B). Indeed, the intensity of the fluorescence signal at the perimembrane area was increased after INDO pre-treatment in response to stimuli (FK, TH or FK + TH), as compared to unstimulated cells (CTRL) (*p* < 0.0001). Conversely, in the absence of INDO pre-treatment, the cells showed an apparent lack of responsiveness to the applied stimuli. These observations emphasize the pivotal role of INDO in sensitizing the cells to the subsequent signaling events, leading to a statistically significant increase of hAQP5 trafficking in perimembrane area in response to stimuli increasing cAMP and calcium second messengers.

### Role of hAQP5 C-terminus in its trafficking

**Fig. 2:**
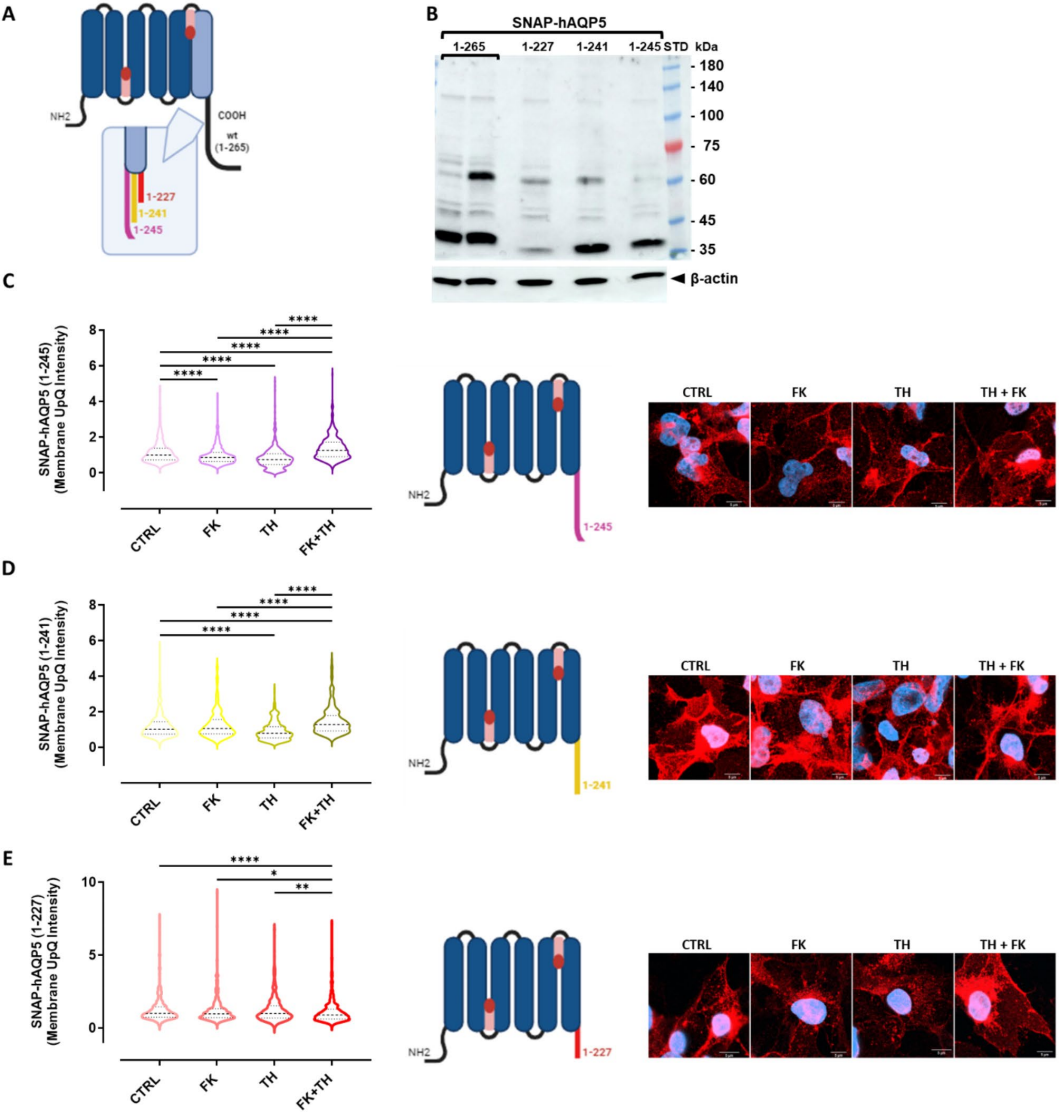
Role of C-terminal domain of hAQP5 in protein trafficking. **(A)** Schematic representations of SNAP-hAQP5 constructs (full length wildtype (1-265)), created with BioRender.com. **(B)** WB analysis for SNAP tag (using anti-SNAP tag antibodies) of NS-SV-AC cells stably transfected with SNAP-hAQP5 (1-265), (1-245), (1-241) and (1-227). STD: molecular weight standards. **(C-D-E-F)** Violin plots (with median and interquartile range of 25–75% percentile) of the membrane upper quartile (UpQ) intensity. Cells were pretreated for 24 h with 50µM indomethacin prior to treatment for 8 h without (CTRL) or with 10 µM FK, 0.1 µM TH, or both (FK + TH), in 3 independent experiments. Statistical significance evaluated using Kruskal-Wallis test with post-hoc Dunn’s tests is indicated as follows: *: *p* < 0.05, **: *p* < 0.01, ***: *p* < 0.001, ****: *p* < 0.0001. Schematic representations of SNAP-hAQP5 constructs were created with BioRender.com. Representative confocal images of immunofluorescent staining with anti-SNAP antibodies (red) and DAPI (blue). **(C)** In SNAP-hAQP5 (1-245), the medians with IQR are: 1 with IQR 0.724–1.375, *n* = 389 cells for CTRL; FK: 0.866 with IQR 0.648–1.158, *n* = 483 cells; TH: 0.644 with IQR 0.171–0.975, *n* = 410 cells and 1.257 with IQR 0.904–1.724, *n* = 428 for FK + TH. **(D)** In SNAP-hAQP5(1-241), the medians with IQR are: 1 with IQR 0.749–1.447, *n* = 431 cells for CTRL; FK: 1.058 with IQR 0.764–1.566, *n* = 535 cells; TH: 0.794 with IQR 0.530–1.160, *n* = 307 cells and FK + TH: 1.290 with IQR 0.927–1.798, *n* = 349 cells. **(E)** In SNAP-hAQP5 (1-227), the medians with IQR are: 1 with IQR 0.750–1.448, *n* = 513 cells for CTRL; FK: 0.974 with IQR 0.713–1.327, *n* = 518 cells, TH: 0.995 with IQR 0.699–1.534, *n* = 503 cells and FK + TH: 0.902 with IQR 0.617–1.296, *n* = 597 cells. The specificity of the anti-SNAP antibodies is shown in Fig. S6A

To assess the role of regions of the hAQP5 C-terminal domain in the protein translocation to the plasma perimembrane area, we generated NS-SV-AC cells stably transfected with truncated SNAP-hAQP5 constructs. The truncations were made immediately after the last transmembrane helix (SNAP-hAQP5 (1-227)), a short structurally conserved cytoplasmic helix that is known to be involved in AQP5 protein-protein interactions (SNAP-hAQP5 (1-241)), or after the last visible residue in the AQP5 crystal structure [35] thus removing the highly flexible distal part of the C-terminus (SNAP-hAQP5 (1-245)) (Fig. 2A). Considering that anti-AQP5 antibodies are directed against the C-terminus end of the protein, it was necessary to perform WB analysis against the SNAP-tag, to confirm the presence of AQP5-immunoreactive bands. WB analysis showed an expected 42 kDa molecular weight for the full-length SNAP-hAQP5 construct (1-265; used as positive control), as well as progressively lower molecular weights for the truncated SNAP-hAQP5 (1-245), SNAP-hAQP5 (1-241), and SNAP-hAQP5 (1-227) constructs (Fig. 2B). The stimuli-induced trafficking of the hAQP5-truncated constructs was then studied following indomethacin pretreatment and incubation without (CTRL) or with FK, TH, or FK + TH, by immunofluorescence using SNAP-tag antibodies. While the trafficking of the SNAP-hAQP5 (1-265) to the perimembrane area was increased by FK, TH and FK + TH (Fig. S7), the construct with a deletion of only 20 amino acid residues from the C-terminal end (SNAP-hAQP5 (1-245)) appears predominantly located in the cytoplasm, with low expression in plasma perimembrane area in the absence of stimuli (CTRL) (Fig. 2C). FK and TH added individually significantly decreased the trafficking of SNAP-hAQP5 (1-245) to the perimembrane area as compared to the CTRL. However, when added together, FK + TH significantly increased the trafficking of SNAP-hAQP5 (1-245) to the perimembrane area (Fig. 2C). Similarly, in the SNAP-hAQP5 (1-241) bearing cells, FK and TH failed to enhance the plasma perimembrane localization, with TH even exerting a significant decrease in hAQP5 levels within the plasma perimembrane area compared to the CTRL cells (Fig. 2D). As for SNAP-hAQP5 (1-245), the combination of both treatments was able to increase significantly SNAP-hAQP5 (1-241) expression at the plasma perimembrane area. In contrast, the SNAP-tagged hAQP5 (1-227) construct, with the most extensive deletion spanning 38 amino acids, exhibited a complete impairment of hAQP5 trafficking upon stimulation, even showing a reduced level of hAQP5 expression under FK + TH (Fig. 2E).

### Involvement of hPIP in SNAP-hAQP5 trafficking

**Fig. 3:**
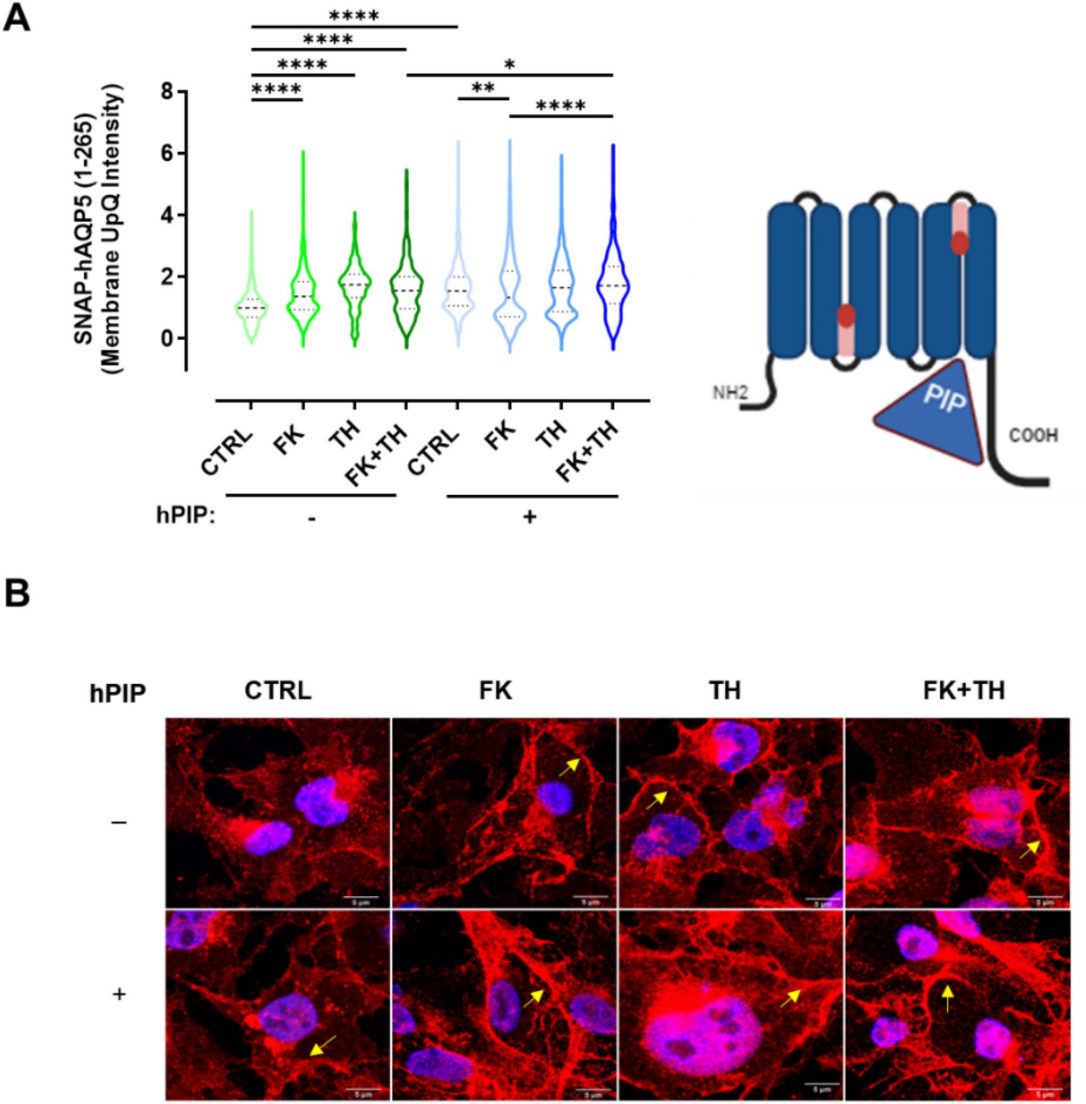
Involvement of hPIP in hAQP5 trafficking. **(A-B)** Cells were pretreated for 24 h with 50µM indomethacin prior to treatment for 8 h without (CTRL) or with 10 µM FK, 0.1 µM TH, or both (FK + TH), in 3 independent experiments. **(A)** Violin plots (with median and interquartile range of 25–75% percentile) of the membrane upper quartile (UpQ) intensity. Statistical significance evaluated using Kruskal-Wallis test with post-hoc Dunn’s tests is indicated as follow: *: *p* < 0.05, **: *p* < 0.01, ****: *p* < 0.0001. In the absence of PIP, the medians with IQR are: 1 with IQR 0.709–1.287, *n* = 482 cells for CTRL; 1.274 with 0.872–1.817, *n* = 721 cells for FK; 1.787 with IQR 1.367–2.192, *n* = 305 cells for TH; 1.493 with IQR 0.890–1.986, *n* = 651 cells for FK + TH. In the presence of PIP, the medians with IQR are: 1.553 with IQR 1.075–2.005, *n* = 606 cells for CTRL; 1.335 with IQR 0.722–2.196, *n* = 596 cells for FK; 1.651 with IQR 0.875–2.216, *n* = 565 cells for TH and 1.717 with IQR 1.136–2.344, *n* = 594 cells for FK + TH. Schematic representations of SNAP-hAQP5 interaction with hPIP was created with BioRender.com. **(B)** Representative confocal images of immunofluorescent staining with anti-AQP5 antibodies (red) and DAPI (blue); yellow arrows indicate AQP5 localization close to the plasma membrane

Considering the previously reported protein-protein interaction between hAQP5 and hPIP, which undergoes alterations in SS patients [27], we evaluated the influence of hPIP on hAQP5 trafficking. To this end, hPIP was stably transfected in NS-SV-AC cells stably transfected expressing SNAP-hAQP5. Indeed, NS-SV-AC cells do not express endogenous hPIP. The expression of hPIP in SNAP-hAQP5 cells was confirmed by immunofluorescence (Fig. S8A). The presence of hPIP increased hAQP5 expression in the perimembrane area in the absence of exogenous stimuli (CTRL; independently of an effect of hPIP on the turnover of the hAQP5 protein (Fig. S8B), as well as in the presence of FK + TH (Fig. 3A, B), as compared to cells that do not express hPIP. Remarkably, in cells transfected with hPIP, the localization of hAQP5 to the perimembrane area was decreased by FK, and unmodified by TH and FK + TH as compared to the CTRL condition (Fig. 3A, B).

## Discussion

The present study investigates the intracellular trafficking of hAQP5 and its regulation, with a focus on the role of the hAQP5 C-terminal tail and of hPIP – an AQP5 interacting protein partner in the acinar salivary gland cell line NS-SV-AC. For this purpose, considering that NS-SV-AC cells are devoid of endogenous hAQP5 and hPIP expression, we successfully generated stably transfected NS-SV-AC cell lines expressing either SNAP-tagged full-length hAQP5 construct (1-265) alone or with hPIP, or a C-terminal truncated SNAP-tag AQP5 construct (1-245; 1-241; 1-227).

We quantified the labeling of SNAP-hAQP5 constructs to the cell perimembrane area using a method accounting for variations in intensity across membrane fragments and conditions. By focusing on the upper quartile intensity, the method ensured that measurements were representative of the true membrane signal, even in the presence of background or cytoplasmic fluorescence. The segmentation process was optimized to balance accuracy with practical constraints, avoiding the need for extensive training of deep learning models. This quantification approach allowed for the comparison of membrane fragment intensities, providing insights into perimembrane activity and localization of the SNAP-hAQP5 constructs under varying experimental conditions. Additional experiments may still be warranted to confirm the localization of hAQP5 at the cell plasma membrane per se using either electron microscopy or double immunofluorescence labeling of SNAP-hAQP5 along with a membrane protein not undergoing any localization changes upon stimuli inducing SNAP-hAQP5 trafficking.

Our data show that the full-length SNAP-hAQP5 (1-265) translocates to the plasma perimembrane area in response to FK, TH or FK + TH stimuli when cells were preincubated with INDO (Fig. 1B). In contrast, cells not pretreated with INDO exhibited an apparent lack of responsiveness to the applied stimuli, underscoring the pivotal role of INDO in inhibiting prostaglandin synthesis and consequently of prostaglandin-induced cAMP and calcium response [36, 37]. Indeed, it can be speculated that INDO treatment allows setting the intracellular levels of the second messengers to a basal level throughout the cell population, sensitizing the cells to stimuli increase second messengers. Consequently, this mechanism results in INDO priming cells to stimuli (e.g. FK, TH, FK + TH) promoting hAQP5 trafficking. These data corroborate the benefit of INDO pre-treatment prior to FK promoting AQP2 trafficking in transfected Madin-Darby canine kidney (MDCK) cells [38]. Furthermore, similarly to the green fluorescent protein (GFP)-tag [12, 39], but with the additional advantage of being significantly smaller (19 kDa versus 28 kDa), the SNAP-tag added to the N-terminus end of the hAQP5 construct does not impede protein trafficking in response to stimuli inducing cAMP or calcium increase. Our data validated our cellular model as a suitable model for studying hAQP5 trafficking using a SNAP-tag construct and further opened avenues for exploring the effects of hAQP5 C-terminal truncations on the protein trafficking. The use of three C-terminal truncated hAQP5 constructs unraveled distinct responses to FK, TH, and their combination. Notably, the mutants with a deletion of 20 and 24 amino acids (SNAP-hAQP5(1-245) and SNAP-hAQP5 (1-241) respectively), exhibiting a reduced response to FK, which stimulates cAMP-dependent pathways (Fig. 2). This emphasizes the importance of the C-terminal domain in the trafficking process, as well as the relative importance of subdomains within the C-terminal domain of AQP5 having distinct secondary structures that may be required for appropriate tertiary structure and/or interaction with protein partners. These results may also emphasize the importance of PKA phosphorylation sites in the distal AQP5 C-terminal region in triggering its translocation to the plasma membrane. These data partially corroborate those showing that hemagglutinin-tagged hAQP5 truncated 20 amino acids from its C-terminus (1-225) was unable to translocate upon activation of cAMP and calcium pathways in HEK293 transfected cells [12]. Literature analysis highlights divergent findings concerning the phosphorylation of specific amino acid residues within the AQP5 C-terminal domain. While in AQP2, exhibiting a high degree of homology with AQP5, the phosphorylation of Ser256 has been well documented to promote protein trafficking to the apical plasma membrane of kidney collecting ducts [40, 41], the corresponding Thr259 site in AQP5 has been shown to be phosphorylated by PKA [18] but not involved in AQP5 trafficking [12, 18]. TH, which raises the cytosolic Ca^2+^-concentration, also promoted the translocation of SNAP-hAQP5 to the plasma perimembrane area (Fig. 1B) whereas SNAP-hAQP5(1-245) and SNAP-hAQP5(1-241) cells exhibited a reduced response to TH (Fig. 2). Interestingly, the unexpected increased membrane localization of SNAP-hAQP5 (1-245) and SNAP-hAQP5 (1-241) upon FK + TH stimulation, compared to the lack of effect of FK or TH alone, suggests that both calcium- and cAMP-dependent pathways may act in synergy on other motifs of the C-terminal tail than those involved in the response to the individual stimuli (FK, TH). This underscores the intricate regulatory mechanisms dictating AQP5 shuttling, which necessitates further exploration to improve the current understanding. Finally, the most extensive deletion of the hAQP5 C-terminus, the SNAP-hAQP5 (1-227), lacking the entire C-terminal tail, unveiled profound alteration in protein trafficking, with a lack of response to FK and TH alone as well as in combination. This most likely indicates a significant disruption of the regulatory mechanisms involving cAMP and calcium. These data highlight the importance of the C-terminal tail, its phosphorylation, structural subdomains and interacting protein binding sites in hAQP5 trafficking involving both calcium- and cAMP-dependent pathways. In the future, additional live cell imaging of hAQP5 trafficking may be warranted taking advantage of the properties of the SNAP-tag to react specifically and rapidly with fluorescent O6-benzylguanine derivatives to label proteins.

Previous studies in mouse lung epithelial cells, naturally expressing AQP5, have reported that cAMP increased both AQP5 mRNA level and AQP5 localization at the plasma membrane [42] However, in transfected NS-SV-AC cells used in this study, hAQP5 is driven by a constitutive non-regulated eucaryotic strong promoter, precluding transcriptional regulation. Notably, short-term exposure (minutes), but not long-term exposure (hours), of mouse lung epithelial cells to cAMP induced AQP5 internalization through a mechanism involving PKA and lysosome-dependent degradation [15]. In this study, at this point, we cannot rule out that the increased localization of the SNAP-hAQP5 at the perimembrane area in response to FK and TH may results not only from trafficking but also regulation at various stages (transcription, translation, post-translation). Nevertheless, effects on transcription, translation, post-translation, increasing SNAP-hAQP5, must be obviously accompanied by an increase in trafficking as well to explain the increased perimembrane localization observed. However, additional studies are warranted to assess the effects of FK and TH on hAQP5 mRNA and protein synthesis and degradation, as well as on hAQP5 mRNA and protein half-life in NS-SV-AC cells transfected with the hAQP5 constructs, to be able to stratify between the aforementioned steps and trafficking.

As mentioned above, the folding of the C-terminal domain may play a role in protein-protein interactions [28]. Indeed, the hAQP5 (1-241) construct ends immediately after a short helix located in the cytoplasm that is formed by the proximal part of the C-terminus and proposed to be a common interaction site for protein partners governing AQP trafficking [22]. Studies of the interaction between AQP2 and the lysosomal trafficking regulator interacting protein-5 (LIP5), have shown that residues beyond this interaction site allosterically control the AQP2-LIP5 interaction in a phosphorylation-dependent manner [43]. Further studies will be needed to evaluate the role of individual amino acids and phosphorylation sites within the distal hAQP5 C-terminus in the trafficking mechanism.

Previous investigation revealed the intricate relationship between hAQP5 and hPIP in salivary glands, providing novel insights into their interaction dynamics and implications in cellular function [27]. PIP is expressed in human and rodent salivary glands [27, 44, 45]. Patients suffering from Sjögren’s syndrome are characterized by lower saliva PIP levels [46] and decreased hPIP expression associated with altered localization of both hAQP5 and hAQP5-hPIP complexes (mostly at the basal plasma membrane instead of apical plasma membrane) in acinar cells from minor salivary glands [27]. PIP knockout mice also displayed altered AQP5 localization in salivary gland acinar cells [27]. These data suggested the involvement of hPIP in hAQP5 trafficking, a key mechanism in saliva secretion. Upon transfection with hPIP, we observed a notable increase in hAQP5 translocation to the plasma perimembrane area in the absence of any stimuli (Fig. 3). This finding underscores the critical role of hPIP in facilitating hAQP5 localization within the plasma perimembrane area. Interestingly, the subcellular localization of hAQP5 was decreased by FK and unaffected by TH or FK + TH when hPIP was expressed; suggesting that overexpression of hPIP attenuates the effect of calcium- and cAMP-dependent pathways on hAQP5 sub-cellular localization. This apparent loss of sensitivity to calcium and cAMP may be due to the basal trafficking level already being significantly increased by the presence of hPIP. It may be useful to label hPIP with a CLIP-tag (also a self-labeling protein derived from human O6-alkylguanine-DNA-alkyltransferase, similar to the SNAP-tag), which reacts specifically with fluorescent O2-benzylcytosine derivatives [47], and perform additional live cell imaging experiments of SNAP-hAQP5 and CLIP-hPIP constructs using the respective SNAP and CLIP fluorescent substrates to further study the role of the hAQP5-hPIP protein-protein interaction on AQP5 trafficking.

## Conclusions

Our study sheds light on the intracellular trafficking of hAQP5 and its regulatory mechanisms involving the C-terminal tail and interaction with hPIP. The development of a robust cellular model, featuring a SNAP-tagged full-length functional variant of hAQP5, enabled in-depth examination of long-term hAQP5 trafficking that could be used in the future for additional high-resolution live cell imaging. The innovative new automated algorithm-based method for evaluating hAQP5 translocation to the plasma perimembrane area upon stimulation provides solid foundation for future investigations of hAQP5 dynamics to explore further the role of specific amino acid residues of the AQP5 C-terminal domain involved in protein-protein interactions and trafficking.

## Electronic supplementary material

Below is the link to the electronic supplementary material.

Supplementary Material 1

## Data Availability

The data that support the finding of this study are available from the corresponding author on reasonable request.

## Abbreviations

AE2: Anion exchanger 2
AQP2: Aquaporin 2
AQP5: Aquaporin 5
AQPs: Aquaporins
cAMP: 3’,5’-cyclic adenosine monophosphate
CLIP: self-labeling protein derived from human O6-alkylguanine-DNA-alkyltransferase
C-terminal: Carboxy-terminal
CTRL: Control
DAPI: 4′,6-diamidino-2-phenylindole, dihydrochloride
ECL: Enhanced chemiluminescence
FK: Forskolin
FL: Full length
GFP: Green fluorescent protein
hAQP5: human aquaporin5
HRP: Horseradish peroxidase
INDO: Indomethacin
IQR: Interquartile range
LIP5: Lysosomal trafficking regulator interacting protein-5
MDCK: Madin-Darby canine kidney
MUC5AC: Mucin 5AC
NKCC1: Na-K-Cl cotransporter 1
NS-SV-AC: Normal salivary gland-SV40 transformed-acinar cell line
PFA: Paraformaldehyde
PBS: 10mM phosphate buffer saline
PBS-T: 10mM phosphate buffer saline with 0.1% Tween 20
PIP: Prolactin-inducible protein
PKA: Protein kinase A
PKG: Protein kinase G
PVDF: Polyvinylidene fluoride
RT: Room temperature
Ser: Serine
SNAP: Small protein derived from O6-alkylguanine-DNA-alkyltransferase
SS: Sjögren’s syndrome
TH: Thapsigargin
Thr: Threonine
TRPV4: Transient receptor potential cation channel subfamily V member 4
UpQ: Upper Quartile
WT: Wildtype
