# Supplementary material for: AQP5 trafficking is regulated by its C-terminal tail and interaction with prolactin-inducible protein
Source: Biol Direct. 2025 Apr 16;20:53. doi: 10.1186/s13062-025-00647-6 (PMC12001509; doi:10.1186/s13062-025-00647-6)
Supplement: Supplementary file 1 — Supplementary Material 1 [file 13062_2025_647_MOESM1_ESM.docx]

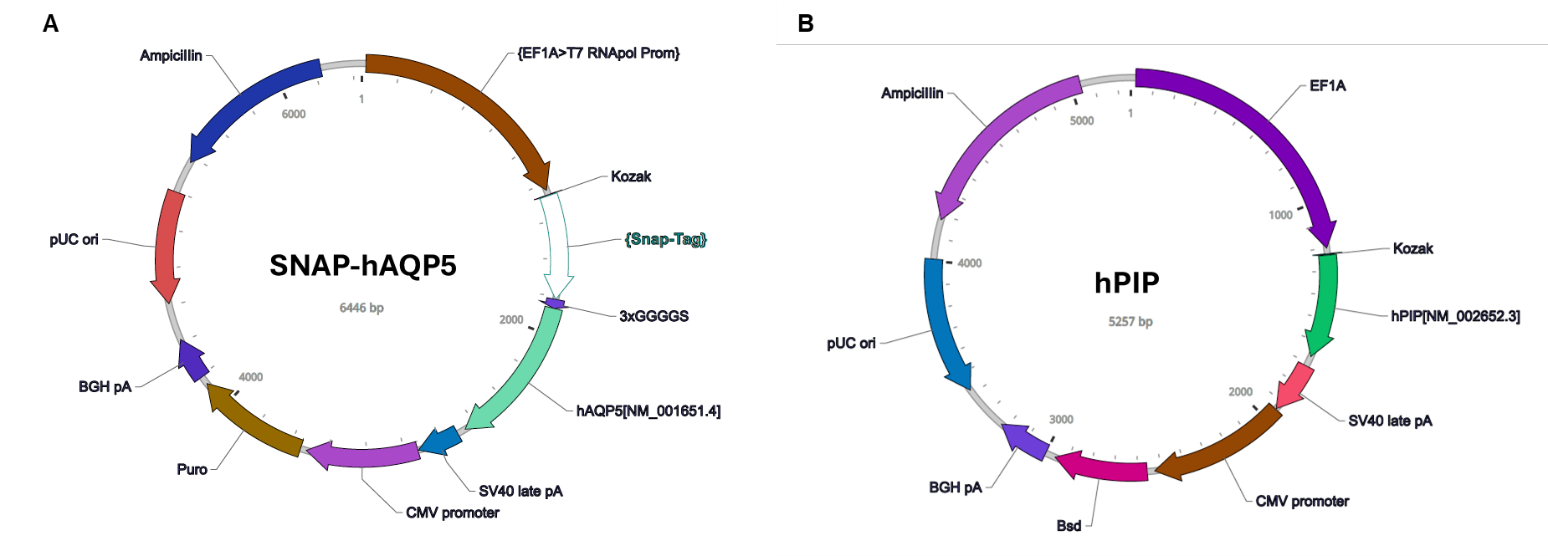


**Figure S1.** **Maps of plasmids.** (**A**) SNAP-tagged hAQP5 (1-265) plasmid. The vector features a robust EF1A promoter followed by a T7 RNA promoter sequence. The promoter drives transcription of a SNAP-tag (a 20 kDa mutant of the DNA repair protein O6 alkylguanine‐DNA alkyltransferase capable of covalently labeling with synthetic probes, specifically benzylguanine derivatives), linked to the amino terminus of hAQP5 by a linker sequence comprised of 3 tandem repeats of GGGGS for enhanced flexibility between the two fusion proteins and reducing steric hindrance. A puromycin (Puro) resistance gene was included to select stably transfected cells (VectorBuilder, Chicago, IL, USA). The C-terminally truncated hAQP5 (1-245), hAQP5 (1-241), and hAQP5 (1-227) were made by introducing a stop codon after position 245, 241 and 227, respectively. (**B**): hPIP plasmid. The vector features a robust EF1A promoter, the prolactin-inducible protein (PIP) coding sequence and a blasticidin (Bsd) resistance gene to allow selection of stably transfected cells (VectorBuilder, Chicago, IL, USA).


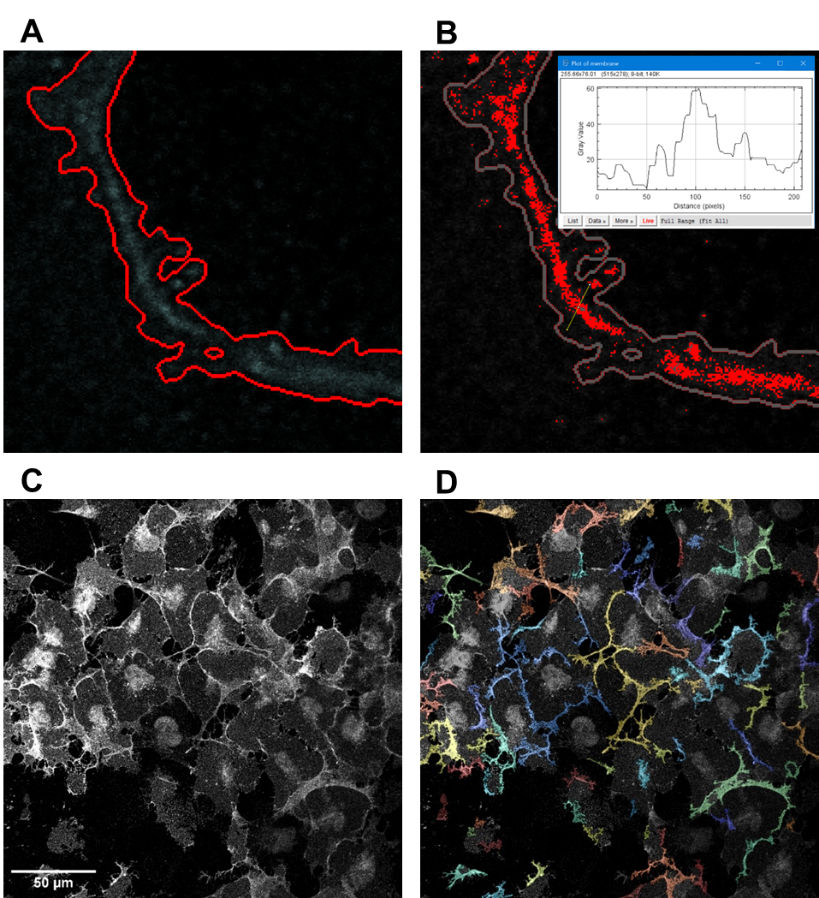


**Figure S2.** **Membrane segmentation.** (**A**) Membrane detection (red outline) may include background pixels due to low signal intensity, as shown by the line profile. (**B**) Segmented pixels with intensity > upper quartile (red pixels, right) are concentrated along the membrane ridge, allowing to isolate key membrane pixels and quantify variations in membrane activity across conditions by analyzing the distribution of upper quartile values across all detected membranes in an image set. (**C**) Example of original image. (**D**) CellProfiler processed image where colors denote individual objects.


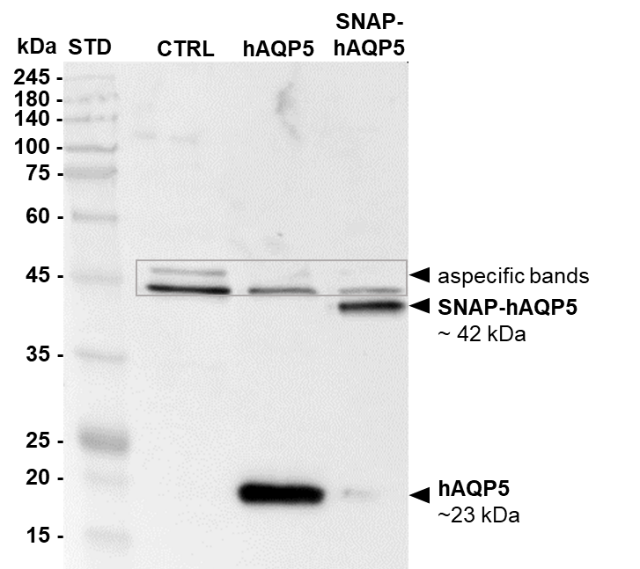


**Figure S3. Expression of hAQP5.** WB analysis for hAQP5 in untransfected NS-SV-AC cells used as negative control (CTRL), and NS-SV-AC cells transfected with tag-free hAQP5 (NS-SV-AC + hAQP5; used as positive control) or SNAP-hAQP5. STD: molecular weight standards.


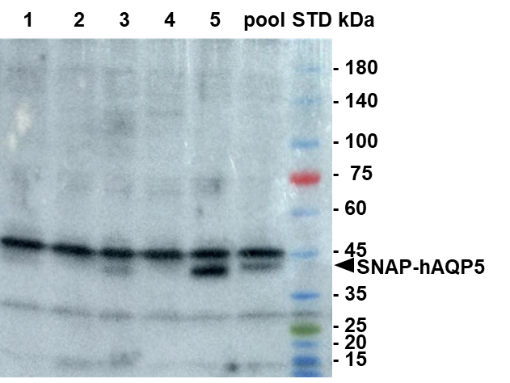


**Figure S4. Expression of SNAP-hAQP5 construct in NS-SV-AC cells.** WB analysis for SNAP-tag in five clones (1-5) or pool (used as positive control prior to limiting dilution cloning) of NS-SV-AC cells stably transfected with SNAP-hAQP5. STD: molecular weight standards.


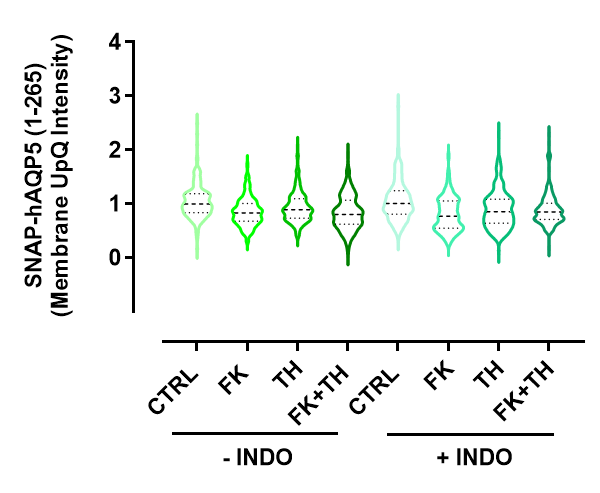


**Figure S5. Trafficking of SNAP-hAQP5 construct in NS-SV-AC cells.** Cells (clone 3 of NS-SV-AC SNAP-hAQP5) were pretreated for 24h without or with 50µM indomethacin (-INDO: +INDO) prior to treatment for 1h without (CTRL) or with 10 µM FK, 0.1 µM TH, or both (FK+TH), in 3 independent experiments. Violin plots (with median and interquartile range of 25-75% percentile) of the membrane upper quartile (UpQ) intensity show that neither FK, TH or FK+TH increased AQP5 trafficking. In the absence of INDO pretreatment, the medians with IQR are: 1 with IQR 0.839-1.190, n=159 cells for CTRL; 0.836 with IQR 0.685-1.010, n=211 cells for FK; 0.898 with IQR 0.740-1.102, n=185 cells for TH; 0.808 with IQR 0.626-1.073, n=226 cells for FK+TH. In the presence of INDO pretreatment, the medians with IQR are: 1 with 0.813-1.246, n=150 cells for CTRL; 0.776 with IQR 0.553-1.059, n=215 for FK; 0.859 with IQR 0.645-1.090, n=192 for TH; 0.854 with IQR 0.714-1.014, n=182 for FK+TH.


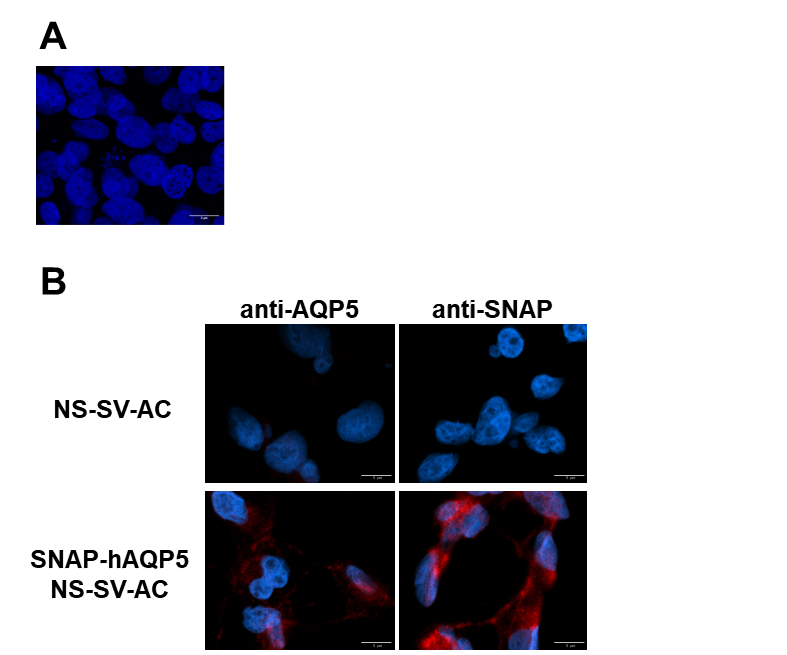


**Figure S6: Negative control and specificity of the antibodies for immunofluorescence.** **(A)** Negative control was performed in the absence of primary antibody. **(B)** Immunofluorescence performed with anti-AQP5 and anti-SNAP antibodies on NS-SV-AC cells and NS-SV-AC cells stably transfected with SNAP-hAQP5 (used as positive control).


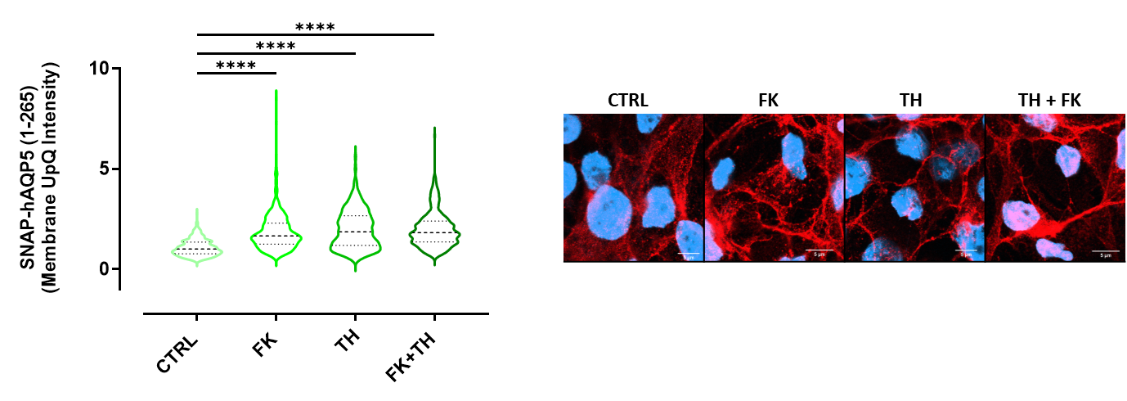


**Figure S7. hAQP5 (1-265) protein trafficking.** Cells were pretreated for 24h with 50µM indomethacin prior to treatment for 8h without (CTRL) or with 10 µM FK, 0.1 µM TH, or both (FK+TH), in 3 independent experiments. Statistical significance evaluated using Kruskal-Wallis test with post-hoc Dunn’s tests is indicated as follows: ****: p < 0.0001. Violin plots shows that the medians with IQR are: 1 with IQR 0.764-1.352, n=379 cells for CTRL; FK: 1.650 with IQR 1.242-2.294, n=523 cells; TH: 1.861 with IQR 1.182-2.659, n=518 cells and 1.813 with IQR 1.361-2.394, n=550 for FK+TH. Representative confocal images of immunofluorescent staining with anti-SNAP antibodies (red) and DAPI (blue).


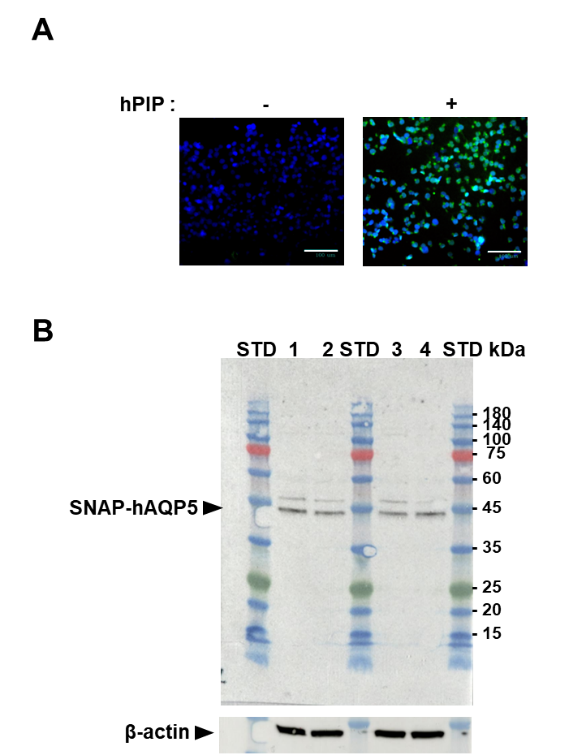


**Figure S8. Expression of hPIP and lack of effect of hPIP on SNAP-hAQP5 expression. (A)** Immunofluorescence for hPIP was performed in clone 3 of NS-SV-AC expressing SNAP-hAQP5 (1-265) transfected without (-) or with (+) hPIP. Representative confocal images of immunofluorescent staining with anti-hPIP antibodies (green) and DAPI (blue); Scale bar of 100µm. **(B)** WB analysis for SNAP-hAQP5 (using anti-SNAP antibodies) and β-actin of two samples of NS-SV-AC cells stably transfected with SNAP-hAQP5 and without (lanes 1, 3) or with hPIP (2, 4). STD: molecular weight standards.
